# A Perspective on Current and Future Metric Tools

**DOI:** 10.1021/acs.analchem.5c04727

**Published:** 2025-10-20

**Authors:** Francisco Pena-Pereira, Juan L. Benedé

**Affiliations:** a Centro de Investigación Mariña, Departamento de Química Analítica e Alimentaria, Grupo QA2, Edificio CC Experimentais, 16784 Universidade de Vigo, Vigo 36310, Spain; b GICAPC Research Group, Department of Analytical Chemistry, University of Valencia, Burjassot, Valencia 46100, Spain

## Abstract

Assessment tools have attracted immense interest in the analytical field in recent years, and remarkable developments have been described toward increasing levels of refinement. However, substantial improvements of metric tools are still needed to obtain highly relevant information about analytical systems in an efficient manner while maintaining or increasing their user friendliness, minimizing their potential subjectivity, and ensuring a high reliability and comparability. The present perspective identifies areas of improvement of currently available metric tools and highlights potential initiatives to be tackled toward advanced metric tools.

## Graphical abstract

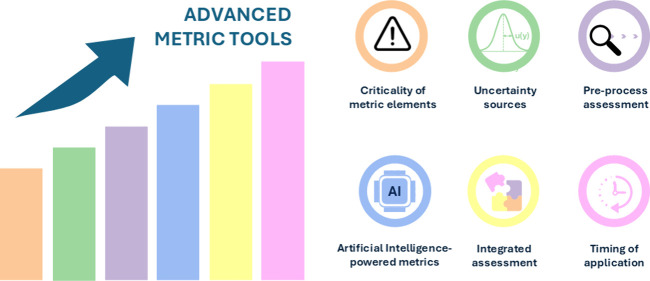

## Introduction

Metric tools have become essential elements in the current research landscape, including the analytical chemistry field. Far from being mere complementary elements, these tools have become fundamental pillars for evaluating the impact of procedures and ensuring that decisions are sound. Well-defined metric tools not only enable one to check the effectiveness of a specific system but also facilitate comparisons between different alternatives, drive informed decision-making, and guide continuous improvement. Furthermore, this role is particularly relevant at a time when analytical chemistry is challenged by the need to analyze complex matrices using increasingly sustainable methodologies, following the principles of green analytical chemistry (GAC),^1^ white analytical chemistry (WAC),^2^ and green sample preparation (GSP).^3^

The existing literature within the analytical chemistry field includes a wide variety of metric tools developed over time with different purposes, mainly derived from the above principles. These tools can be classified according to their primary focus, i.e., the assessment of the overall attributes of the system (e.g., RGB model^4^ and its expansions (RGB12 ^2^ and RGBfast^5^) and Hexagon-CALIFICAMET^6^), or only some of its aspects according to the WAC concept, namely, the analytical performance (i.e., quality of the results) (e.g., Red Analytical Performance Index (RAPI)^7^), the practicality and viability of the system (e.g., Blue Applicability grade Index (BAGI)^8^), or the safety and the environmental impact (e.g., National Environmental Methods Index (NEMI),^9^ Green Analytical Procedure Index (GAPI)^10^ and its variants,^11–13^ and the Analytical Greenness Calculator (AGREE)^14^), the latter being the most abundant to date. Recently, a tool has been designed to assess the degree of innovation of analytical systems to complement the above red, blue, and green metrics (i.e., Violet Innovation grade Index (VIGI)^15^). Figure 1 shows a timeline of the most relevant metric tools along with milestones in the field of analytical chemistry.

**Figure 1:**
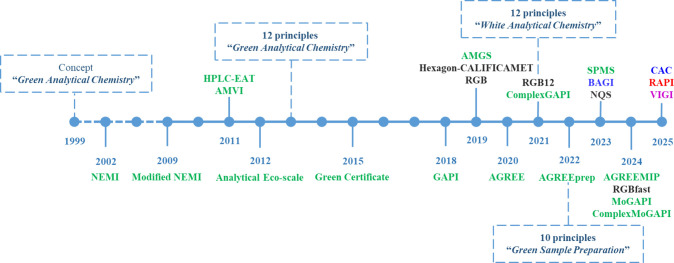
Timeline of the most relevant metric tools along with the milestones in the field of analytical chemistry. (AGREE: Analytical Greenness calculator; AGREEMIP: Analytical Greenness Assessment Tool for Molecularly Imprinted Polymers Synthesis; AGREEprep: analytical greenness metric for sample preparation; AMGS: Analytical Method Greenness Score; AMVI: analytical method volume intensity; BAGI: Blue Applicability Grade Index; CAC: Click Analytical Chemistry Index; GAPI: green analytical procedure index; HPLC-EAT: high-performance liquid chromatography-Environmental Assessment Tool; NEMI: National Environmental Methods Index; NQS: need, quality and sustainability; RAPI: red analytical performance index; RGB: red-green-blue; SPMS: sample preparation method for sustainability; VIGI: violet innovation grade index). The font color indicates the type of metric based on its focus (red: analytical performance; green: safety and environmental impact; blue: practicality and viability; violet: innovation; black: overall attributes).

A second axis of classification for these tools can be established based on the stage of the analytical process (i.e., sampling, sample preparation, and determination) for which their application is designed. In this sense, most metric tools are applicable to assess the entire procedure from the point of view of the different dimensions of WAC (i.e., general metrics), while others are specific to evaluate certain stages, such as Analytical Greenness Metric for Sample Preparation (AGREEprep)^16^ and Sample Preparation Method for Sustainability (SPMS)^17^ for the assessment of the sample preparation stage and Analytical Method Volume Intensity (AMVI),^18^ HPLC-Environmental Assessment Tool (EAT),^19^ and Analytical Method Greenness Score (AMGS)^20^ for evaluating chromatographic separations. Additionally, some specific tools have been presented to evaluate the impact of the solvents and reagents used in analytical procedures (e.g., ChlorTox Scale^21^) as well as in the preparation of materials for analytical purposes (e.g., Analytical Greenness Assessment Tool for Molecularly Imprinted Polymers Synthesis (AGREEMIP)^22^).

In this regard, it is undeniable that significant progress has been made in the design and implementation of a variety of increasingly refined metrics into the analytical workflow. However, this plurality of approaches, although enriching, also poses certain challenges, such as the coexistence of metrics with different levels of maturity, which can make an effective comparison between studies difficult. This highlights the fact that the path to truly advanced or next-generation metric tools is still under construction.

It should be noted that several reviews have already described these metric tools in detail, analyzing their advantages, limitations, and specific areas of application.^23,24^ Additionally, very recently, Tobiszewski et al.^25^ and Nowak^26^ have proposed strongly recommended guidelines and general rules for the implementation of good evaluation practice to follow for a correct selection and application of metric tools toward more reliable assessments, and Fuente-Ballesteros et al.^27^ have proposed 10 principles to evaluate different attributes (practical, reproducible, inclusive, sustainable and manageable, PRISM) of the metrics themselves that promotes clarity, usability, and consistency in their development. Therefore, the purpose here is not to repeat this exhaustive analysis but rather to offer a perspective that highlights current trends and persistent challenges. The following sections propose a reflection that acts as a roadmap to identify areas for improvement, propose more integrative design criteria, and point out potential initiatives that will help overcome some of the current limitations. Ultimately, the goal is to pave the way for a more holistic approach to the design and application of advanced metric tools aligned with current and emerging demands.

## Challenges and Potential Initiatives Toward Improved Metric Tools

### Considerations on the Criticality of Metric Elements

Different metric elements are required for the evaluation of the analytical systems. Particularly, the number and type of criteria, the functions employed for the assessment of individual criteria, and the weights selected for obtaining an overall result of assessment are of paramount importance in the evaluation of analytical systems. Thus, considerations regarding the criticality of metric elements for the application of current metrics and the development of future metric tools (or refined new versions of existing ones) are provided below.

#### Type and Number of Criteria

The type and number of criteria considered in metric tools are highly variable, and both aspects can affect the assessment of analytical systems. The number of criteria included in metrics has increased from the four criteria considered by NEMI to more than 20 criteria in more recently reported metric tools. The selection of criteria must ensure the representativeness of the analytical systems under assessment in terms of the purpose of the metric tool (i.e., general or specific information). However, it should be taken into account that, as in method development, not all variables (i.e., criteria) considered in metric tools necessarily show a significant effect on the response (i.e., score or assessment result). Therefore, the inclusion of criteria that show little or negligible impact in the assessment can substantially increase the evaluation time and distort the overall evaluation result if inappropriate weights (discussed in the *Weights* section) are selected. In general, relevant criteria that are unambiguous and well-defined should be considered whenever possible to facilitate the data collection process and minimize the potential inconsistencies associated with both source data and user interpretation. In this regard, the use of criteria based on directly measurable empirical data is highly recommended, as discussed elsewhere.^26^ Potential indicators of this kind could include the carbon footprint associated with the analysis of a certain number of samples, the total volume of water (e.g., tap, distilled or ultrapure) required by a given method, or the amount of electricity required to perform a certain number of analyses, to mention some examples proposed in the literature.^26^ Nevertheless, criteria that could not be sufficiently specific to be unequivocally interpreted by users should not be omitted for this reason if they are relevant for the assessment (e.g., degree of automation). A recent contribution demonstrates that the overall results obtained with more than a dozen of currently available metric tools show a non-negligible and variable reproducibility, being partially associated with the subjective elements considered in each metric tool.^25^ In this regard, it might be interesting to identify, estimate, and provide the uncertainty associated with each individual criteria of metric tools, as discussed in the Consideration of Uncertainty Sources in the Assessment section. Besides, the assumption of independence of the criteria included in the metric tools could be incorrect in certain cases, and thus, the overall assessment could also be influenced by the potential interactions between relevant interdependent criteria.^28^ Identifying potential redundancies and establishing adjusted weights could be required in these cases to avoid or minimize the bias.

#### Weights

As discussed above, the relevance of the criteria considered in the assessment can be highly variable, and therefore, suitable weights should be employed to transfer the corresponding levels of importance of the criteria to the overall assessment result. The overall performance of an analytical system is thus critically dependent on the weights applied to each criterion, although the comparability of the individual results obtained by the assessment of a number of analytical systems is ensured as long as the evaluation results of individual criteria are also provided. It is striking to note that most currently available metric tools do not explicitly consider weights, in spite of their importance in the overall assessment of analytical systems, or alternatively, equal weights are expressly assigned to the decision criteria. In both cases, this means, in practice, that all factors are considered to be of equivalent relevance in the assessment involving these metric tools. This is mainly, but not exclusively, the case in metric tools whose output is visual (with color scale) but not numerical (e.g., NEMI or the original GAPI tool). Alternatively, some metrics (e.g., analytical ecoscale and SPMS) implicitly assign different importance (i.e., weights) in the assessment by considering different individual scores (or penalty points) depending on the criterion assessed, whereas adjustable weights (for which default values are initially set) can be selected by the users in other metric tools (e.g., AGREE and AGREEprep). The latter option offers the users the possibility to modify the weights depending on the purpose of the assessment, bearing in mind the peculiarities of the evaluated analytical systems. Default weights are, however, widely selected in the contributions employing metric tools that provide this option to the users. In light of the above, *a priori* generally acceptable and justified weights that could still be modified when a given application requires the modification would be a suitable option. Identifying default weights that could be considered valid for a majority would thus be highly recommendable. This can be, for instance, carried out by involving a sufficiently large number of experts in the field to evaluate and establish the relative importance of criteria. Examples on the selection of weights based on the users’ expertise can be found elsewhere.^29^ Alternatively, the establishment of unbiased and objective criteria weights in metric tools without the need to resort to expert judgment^28^ is another potentially feasible option that remains unexplored in the development of metric tools for the evaluation of analytical systems.

#### Boundaries and Functions

The assessment of individual criteria is commonly carried out by establishing ideal and nonideal conditions (i.e., acceptable and unacceptable levels of the criteria) and assigning them the corresponding output values (i.e., scores or pictogram color codes). By way of example, NEMI establishes that a method yielding a waste amount equal or lower than 50 g could be considered as acceptable in terms of waste generation per sample (i.e., quadrant filled-in with green color), whereas a method could be considered as “less green” if the amount of waste generated is larger than 50 g (i.e., quadrant left blank).^9^ Therefore, the boundary for waste generation is established at 50 g in this metric tool. A wide variety of options are considered for the evaluation of individual criteria after establishing the boundaries, ranging from simple yes/no binary responses (e.g., NEMI) to more discriminating functions that can take any value (result) within the corresponding interval, although three- and four-level responses (i.e., staircase functions with three and four intervals) are mainly employed in metric tools. Figure 2 exemplifies this for the evaluation of waste generated in a single analysis (Figure 2a–e) and exclusively in the sample preparation step (Figure 2f,g). Even though the selection of a finite number of options is mandatory for discrete variables (e.g., degree of automation) and can be fairly acceptable for continuous variables (e.g., amount of waste generated in the analysis), the highest degree of refinement is achieved with the highest possible number of levels for discrete variables and functions that can yield multiple values for the evaluation of continuous variables. With respect to the latter, different options with their own pros and cons have been considered for the evaluation of individual criteria between their corresponding ideal and nonideal boundaries. Thus, the relationship between the evaluated criterion and the corresponding output can be modeled with a linear function, whereas alternatives such as logarithmic or exponential functions have also been explored to foster the adoption of, e.g., greener options. Regarding this, the functions applied for the assessment of certain criteria deserve particular attention. By way of example, a three-level scale set by Raynie and Driver in the late 2000s^30^ has been adopted (or adapted) in different metric tools for the assessment of energy consumption (e.g., analytical ecoscale, AGREE). Although this performs reasonably well at the basic level of application, the importance of energy efficiency in the assessment of any process (and therefore also the analytical process and their specific steps) makes it necessary to supplement and improve the inventory of equipment while considering more realistic and discriminative functions to define energy consumption. In this vein, few initiatives have been recently taken to establish more accurate estimations of energy demands of apparatus employed in analytical laboratories^20,31,32^ and could be considered toward improved metrics.

**Figure 2:**
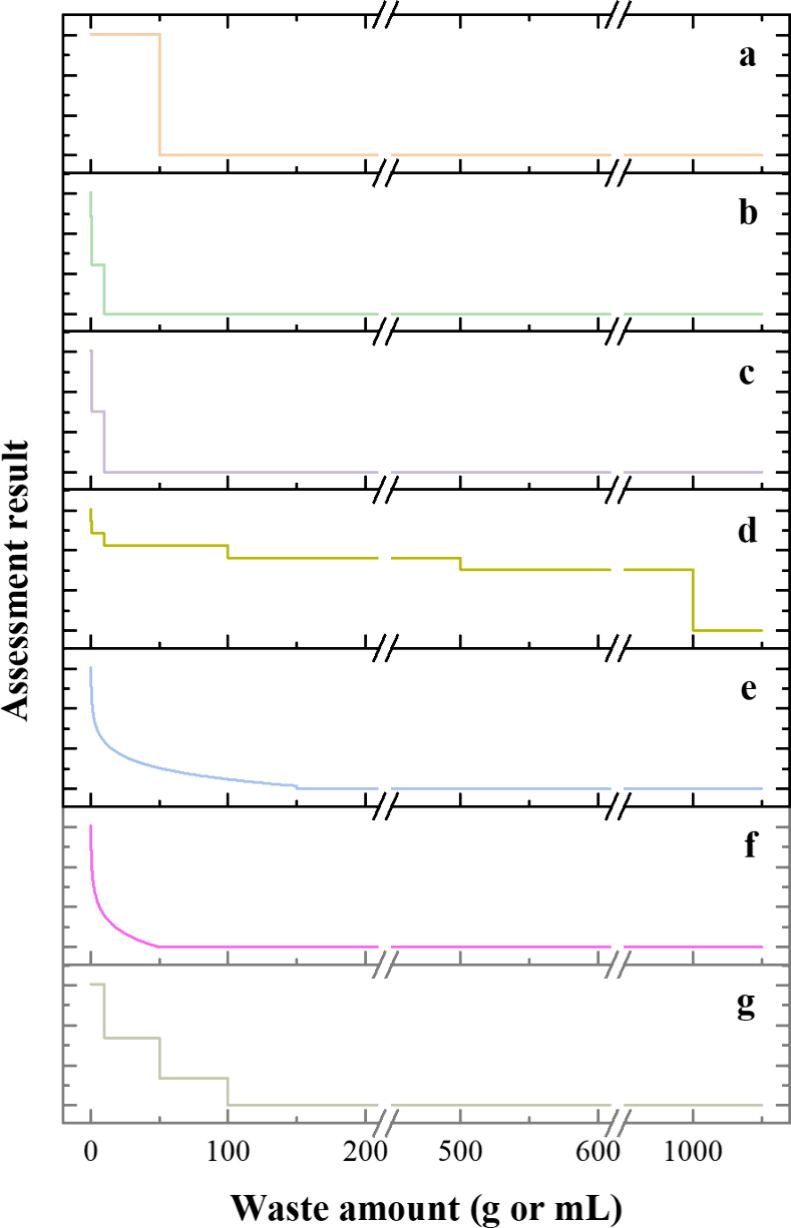
Effect of the waste amount on the assessment results of general-purpose metrics (namely, NEMI (a), analytical ecoscale (b), GAPI (c), HEXAGON-CALIFICAMET (d), and AGREE (e)) and specific metrics (namely, AGREEprep (f) and SPMS (g)).

It should be noted that the criteria were evaluated primarily individually. However, there may be interactions between two (or more) criteria (e.g., amount and harmfulness of a solvent), and therefore, it may be appropriate to use two-factor response surfaces to evaluate two variables simultaneously.

The selection of ideal and nonideal boundary conditions is particularly critical in the development of metric tools. In fact, boundaries that are excessively wide or narrow can insufficiently discriminate between the evaluated criteria at certain levels. The boundaries for each criterion should be carefully selected considering the levels assumed as ideal and nonideal in both conventional and cutting-edge approaches. Moreover, the functions employed both within and outside the boundaries should preferably be preset to avoid potential discrepancies in the assessment associated with the subjective perspective of the evaluators.^33^ It should also be taken into account by users and developers that, thanks to the ongoing development of analytical systems toward greener practices, both the ideal and nonideal boundary conditions set for a given criterion in a metric tool can be progressively shifted. It is therefore advisible to regularly evaluate their applicability and proceed to their refinement in case they are considered out of date, so that the metric tools can be updated and adapted effectively over time.

### Consideration of Uncertainty Sources in the Assessment

Uncertainty is a key concept in metrological sciences that, however, has not received the required attention in the metric tools employed for evaluating analytical systems. The term uncertainty is defined as “a parameter associated with the result of a measurement, that characterizes the dispersion of the values that could reasonably be attributed to the measurand”.^34^ Thus, providing an estimate of uncertainty associated with the quantity being measured (i.e., result of assessment) would increase the reliability of the assessment with regard to the regular practice of considering the measured value with ultimate certainty.

Identifying the main criteria that can lead to uncertainty in the obtained scores would thus be particularly useful for improved assessment of analytical systems. In some cases (e.g., in official methods of analysis), flexible experimental conditions (e.g., sample amount) can be used for a given analysis without affecting the methods’ performance.^35^ This fact is absolutely favorable in terms of its robustness and adaptability to a given analytical problem. However, the lack of specific conditions leads to certain input data variability that increases uncertainty in the assessment. Additionally, some criteria are particularly prone to interpretation variability among different users, such as energy consumption, which is directly dependent on the equipment; the amount of waste generated; or the actual times involved in each stage of an experimental procedure, which directly affects the throughput of the method. In this sense, assumptions are commonly made to assess analytical systems with previously reported metrics, even though the availability of metric tools that consider uncertainty contributions would be particularly informative. Thus, it would be desirable to identify (at least) the main contributions to the uncertainty associated with individual criteria and preferably provide them in advanced metrics. As in the estimation of uncertainty in analytical measurements, smaller contributions to uncertainty can be considered negligible for estimating the uncertainty of the assessment scores, as the largest contributions would represent good estimates. In addition, the overall assessment results should include a combined uncertainty estimation by adopting uncertainty propagation principles to establish the confidence of the assessment results.

Moreover, it is worth noting that metric tools are commonly employed to compare analytical systems according to their performance. However, comparisons are typically carried out by considering the results derived from the assessment without a clear means to discriminate between the evaluated analytical systems. Thus, the estimation of the uncertainties discussed above would also be helpful to statistically determine whether methods evaluated with a given metric tool significantly differ from a given level of significance.

### Applicability of Preprocess Assessment Tools

Current metric tools focused on GAC and GSP hardly consider materials and steps required before the evaluated system, i.e., the analytical process and sample preparation process, respectively. With very few exceptions,^11^ the reagents, solvents, energy, and consumables required for the preparation of lab-made materials to be employed in an analytical method are typically omitted in the assessment, thus neglecting their potential environmental, health, and/or safety impact. The development of assessment tools that take into account previous stages could thus be helpful in identifying commonly overlooked issues. However, data gathering can be particularly complicated and time demanding, and defining the system boundaries is needed to ensure comparability. In this vein, currently available preprocess assessment tools such as ComplexGAPI (and its refined quantitative counterpart, ComplexMoGAPI) consider the production of reagents, solvents, and other materials prior to their use in a given analytical method, but it might also be necessary to extend the assessment to other commercially available materials employed in different steps of the analytical process (e.g., extraction cartridges and analytical columns). It should not be omitted, however, that the assessment could be increasingly complex and challenging if also considering the synthesis steps required for obtaining the intermediate chemicals needed for the preparation of a given material employed in the analytical process. For instance, the synthesis of 1-butyl-3-methylimidazolium tetrafluoroborate, a common ionic liquid that requires more than 30 steps to be prepared,^36^ can be considered to exemplify the complexity of this approach.

### Automatically Generated Assessment Output

Evaluating the overall attributes of an analytical system with current metric tools involves manually entering data and interpreting the outputs based on predetermined criteria. Manual data entry, while useful, is time-consuming, requires expert knowledge, and is prone to inconsistencies between different assessments and, even more, between different assessors, as discussed in the Consideration of Uncertainty Sources in the Assessment section. Therefore, a paradigm shift is anticipated toward assessments that no longer depend directly on the values entered by the user. Instead, the integration of artificial intelligence (AI) into metric tools could be a revulsive as in other scientific and analytical areas.^37–39^ Thus, metric tools that incorporate advanced text analysis capabilities to automatically extract relevant information from scientific documents would be of major significance. This would not only make assessments more accessible to all researchers regardless of their expertise in metric tools but also reduce uncertainty sources (i.e., subjectivity) and standardize evaluations, which will promote more comparable results within the scientific community. AI has recently been applied to assist in the quantification of greenness of chemical syntheses.^40^ The authors report experimental attempts using ChatGPT trained with the ChlorTox Scale, highlighting both its potential (speed and flexibility) and limitations (errors, hallucinations, and need for expert oversight). Alternative approaches involving expert-guided AI training, consensus-driven models, and explainable AI are proposed.^40^ These early experiments demonstrate that the integration of AI models into metrics tools is promising but is still in its early stages.

On other hand, these metric tools could store historical evaluation data, allowing users to access previous assessments and use them as templates for new evaluations. This approach would be especially useful when adapting or modifying existing methods for new analytes or matrices. In these cases, users could apply small changes to the method and immediately assess how those changes affect the assessment without restarting the entire evaluation process, which can sometimes be tedious. Moreover, a crucial evolution of this process is the creation of shared and collaborative databases.^40^ Following the current trend of open access content, these databases would allow assessments to be made public or shared among interested researchers, facilitating transparency, accessibility, and knowledge accumulation. Over time, this collaborative approach could generate a substantial data set that not only reflects the overall potential of analytical systems but also facilitates comparative studies.

Looking ahead, this accumulated data could pave the way for a new generation of tools that assist in the optimization of the method, instead of simply evaluating the attributes of the final method (i.e., *ex post* evaluation).^41^ These tools could recommend experimental conditions that align with whiteness scores based on previous assessments of similar analytes or applications, as also proposed for synthetic greenness.^40^ For this, these new tools incorporate an input parameter for the type of analyte or application being studied. This would drastically accelerate method development by identifying optimal conditions early in the workflow. As an example, if previous assessments have demonstrated that a particular solvent consistently achieves a high degree of compliance with WAC dimensions for similar applications, then the system could recommend those approaches. These advanced tools would be in line with the machine learning (ML) concept, which analyzes big data sets.^42^ ML algorithms learn from historical data to extract valuable information and enables to predict the behavior of unknown analytical systems, thereby offering new opportunities for improving decision-making.^43^ In fact, this approach is already being applied in several fields, including the prediction of chromatographic conditions.^44^

### Integration of Criteria and Metrics

The numerous current metric tools in analytical chemistry, each with its pros and cons, typically operate independently and reflect only a portion of the overall impact of the method. As described in the Introduction section, some tools focus mainly on environmental aspects (e.g., toxicity, waste generation), others on economic factors (e.g., productivity and cost), and still others on analytical performance (e.g., sensitivity, reproducibility). This fact forces researchers to consider the use of more than one tool for assessing their systems. Therefore, the frontier to overcome is to unify the different visions of metrics in integrated frameworks so that they provide a more holistic view of the overall attributes of the method. This integration would allow users to compare systems on a multidimensional scale and recognize synergies between environmental, economic, and analytical criteria. By way of example, a method that is slightly less sensitive (even though suitable for the intended use) but significantly reduces toxicity and energy consumption would be a method in line with the WAC principles. Some available metric tools already cover these three dimensions of WAC (e.g., RGB, Hexagon-CALIFICAMET, NQS), even though there is still a necessity to enhance their accessibility for all users. The implementation of socio-economic and/or environmental-societal indicators in metric tools, while challenging, might be of relevance toward a holistic evaluation of analytical methods in terms of environmental impact, economic development, and societal equity.^45^ The economic impact of analytical methods is partially considered in a few metrics, but this dimension still requires substantial improvement. It should be borne in mind, however, that the cost of materials, energy, etc. is largely dependent on the geographic location, and therefore, the evaluation of the economic impact of analytical systems requires establishing harmonized conditions so that comparability is not impaired. The social implications of analytical methods are wide-reaching, but the assessment of their social relevance can be highly subjective, dependent on the aim of the analysis and even on the geographic location. In fact, the perception of social impact can vary greatly, and importantly, the social impact of a given analytical method might not be unique, as a given method could be employed to solve different analytical problems with highly variable social impacts. Environmental-societal metrics have in fact been considered for the assessment of analytical methods (e.g., the NQS metric tool) in an incipient manner. Particularly, the agreement of evaluated methods with the 17 sustainable development goals (SDGs) set by the United Nations has been considered for the assessment of a “sustainability” attribute. This approach is certainly valuable, even though not all SDGs are directly applicable to analytical methodologies or, at least, the impact that a certain analytical method can have on the achievement of some of these goals (e.g., gender equality or peace, justice, and strong institutions) might be marginal in comparison with others, and the agreement with each SDG is interpretive. The search and implementation of alternative, less user-dependent indicators would be helpful for holistic assessments.

In addition to these comprehensive and unified tools, there is also a challenge in developing more specific tools to evaluate general or specific aspects of particular interest in the analytical field for which there are no perfectly adequate or aligned metric tools for their evaluation, as was the case until recently, for example, with the synthesis of MIPs.^22^ Thus, in this same direction, new tools may be necessary to evaluate the synthesis of other materials widely used in analytical chemistry.

Additionally, there is a need to continually improve and update the criteria. As mentioned above, advances in analytical systems in terms of toxicology, regulatory issues, or instrumentation can quickly shift the boundaries associated with certain criteria. Therefore, incorporating these changes into the metric tools ensures that they remain relevant and align with current societal and scientific needs. As a result, these improvements must be accompanied by flexible tools that allow them to be modified based on user needs or the regulatory context. As a general example, an effective strategy would be to incorporate profiles so that the user could select an “academic mode”, where environmental criteria could receive greater weight, or an “industrial mode”, where productivity and analysis costs are commonly prioritized. This flexibility would allow to use the same metric tool, adapted to the user’s profile, to evaluate analytical systems according to both profiles in a customized but comparable manner without compromising the validity of the analysis.

Likewise, these advanced tools could incorporate adaptation modules for different geolocation contexts. As an example, a method designed in Europe could be adapted to regulations in another part of the world, thus assessing not only the environmental impact but also the practical feasibility.

At this point, it should also be highlighted that many of the metrics developed to date have emerged from the continuous interaction between developers and end-users. For instance, the original GAPI tool evolved into refined versions aimed at more comprehensive (e.g., ComplexGAPI) and quantitative (e.g., MoGAPI and ComplexMoGAPI) assessments of analytical methods that in part reflect the constructive dialogue between developers and practitioners and the evolutionary trajectory of widely used metric tools. In fact, active communication between both stakeholders facilitates the identification of gaps and offers invaluable opportunities for the improvement of existing tools and even for the development of novel advanced metrics.

### Timing to Apply the Metric Tools

One of the main shortcomings of current practice is the ultimate application of the metric tools in analytical flow. In most cases, metric tools are only applied after the analytical method has been fully developed, validated, and even applied to real-world samples (i.e., *ex post* evaluation). At this point, the method is typically considered finalized, and regardless of the evaluation results (whether favorable or not), the conditions of the method are rarely modified. This approach reduces the role of assessment to a confirmatory exercise rather than a constructive tool.

To truly advance toward the ideas that sustainable analytical chemistry promotes, it is essential to apply metric tools during the early stages of method development (i.e., *ex ante* evaluation),^41,46,47^ especially during the optimization of experimental variables. In this way, researchers can make more informed decisions that not only optimize analytical performance, which undoubtedly must be maintained, but also minimize the environmental impact and improve the productivity. For example, when selecting a solvent, current practices typically focus exclusively on comparing the analytical performances obtained between several tested solvents. However, by incorporating these metric tools earlier, researchers could also consider toxicity and other parameters from the assessment output. In short, the goal would be to identify the solvent that balances analytical performance (ensuring that the method is “fit for purpose”) with environmental friendliness. Similarly, the optimization of a time (e.g., extraction time or reaction time) could consider not only which value provides the highest sensitivity but also which one allows for the highest sample throughput (i.e., high productivity) or minimizes energy consumption, among other factors.

Finally, sustainability concepts should be incorporated into university curricula and laboratory practices through the use of metric tools. This provided students with a proactive and sustainable mindset from the beginning. This early integration is crucial to fostering a new generation of analytical chemists who are not only specialized in (bio)­chemical measurement processes but also conscious of environmental responsibility.

## Concluding Remarks

This perspective addresses critical considerations on the assessment of analytical systems, identifying challenges and potential pathways toward advanced metrics and being of potential relevance for both users and developers. The contribution provides insights into the crucial role of metric elements that could be valuable for the conscious application of assessment tools. In addition, an in-depth diagnosis of areas of improvement is undertaken and new initiatives are identified (Figure 3), some of them still very incipiently considered, laying the foundations for the development of advanced metric tools. In this regard, it is not the aim of this perspective to provide a unique vision (that of the authors) but to provide potential solutions that could assist metric developers to significantly refine and/or improve metric tools in very different ways and with varying degrees of implementation.

**Figure 3:**
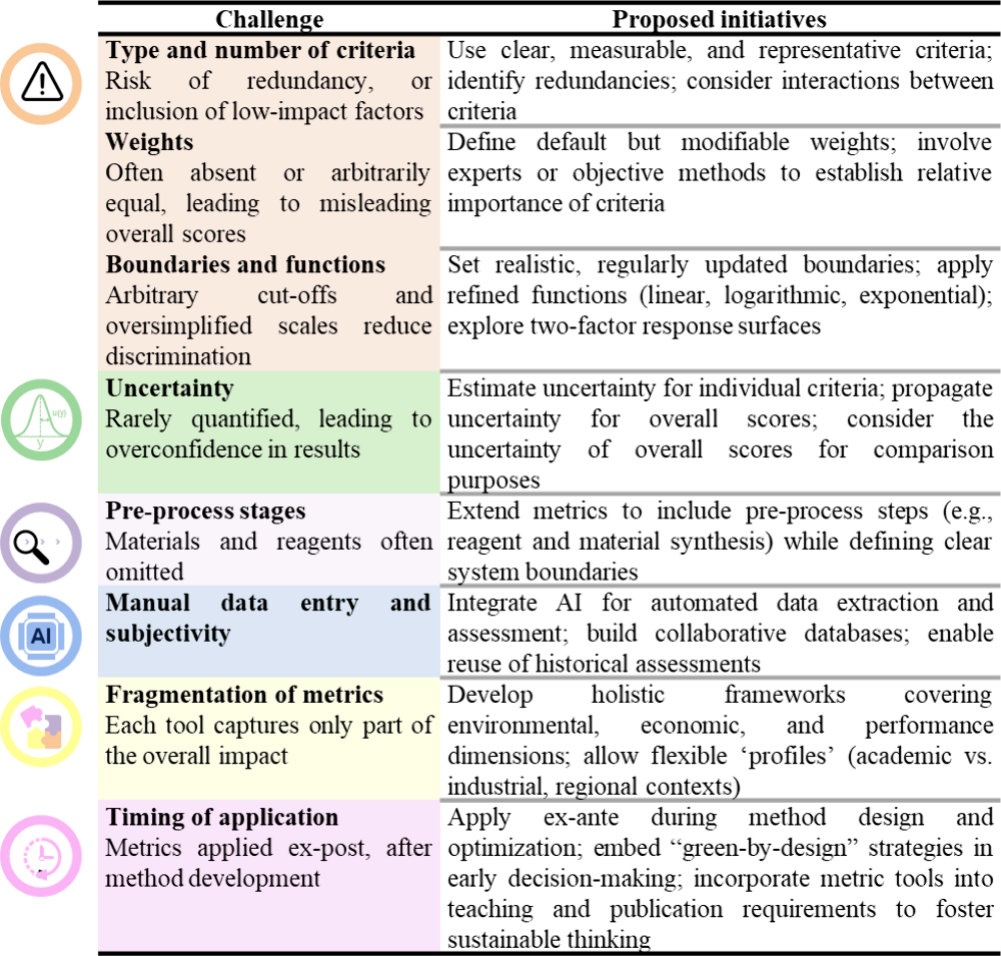
Challenges and potential initiatives toward advanced metric tools.

The authors strongly advocate enhancing existing tools through refined versions rather than developing entirely new ones that do not represent a significant advance over the metrics already available, except when groundbreaking and innovative solutions and viewpoints are considered, several of which are outlined or can be derived from this perspective.
